# Effect of Addition of Natural Antioxidants on the Shelf-Life of “Chorizo”, a Spanish Dry-Cured Sausage

**DOI:** 10.3390/antiox4010042

**Published:** 2015-01-14

**Authors:** Mirian Pateiro, Roberto Bermúdez, José Manuel Lorenzo, Daniel Franco

**Affiliations:** Centro Tecnológico de la Carne de Galicia, Rúa Galicia No. 4, Parque Tecnológico de Galicia, San Cibrao das Viñas, 32900 Ourense, Spain; E-Mails: mirianpateiro@ceteca.net (M.P.); robertobermudez@ceteca.net (R.B.); jmlorenzo@ceteca.net (J.M.L.)

**Keywords:** dry-cured chorizo, natural antioxidants, physicochemical parameters, microbial counts, lipid oxidation

## Abstract

The dose effect of the addition of natural antioxidants (tea, chestnut, grape seed and beer extracts) on physicochemical, microbiological changes and on oxidative stability of dry-cured “chorizo”, as well as their effect during the storage under vacuum conditions was evaluated. Color parameters were significantly (*p* < 0.05) affected by the addition of antioxidants so that samples that contained antioxidants were more effective in maintaining color. The improving effects were dose-dependent with highest values with the dose of 50 mg/kg during ripening and depend on the extract during vacuum packaging. Addition of antioxidants decreased (*p* < 0.05) the oxidation, showing thiobarbituric acid reactive substances (TBARS) values below 0.4 mg MDA/kg. Natural antioxidants matched or even improved the results obtained for butylated hydroxytoluene (BHT). Regarding texture profile analysis (TPA) analysis, hardness values significantly (*p* < 0.001) decreased with the addition of antioxidants, obtaining the lower results with the dose of 200 mg/kg both during ripening and vacuum packaging. Antioxidants reduced the counts of total viable counts (TVC), lactic acid bacteria (LAB), mold and yeast. Free fatty acid content during ripening and under vacuum conditions showed a gradual and significant (*p* < 0.05) release as a result of lipolysis. At the end of ripening, the addition of GRA_1000_ protected chorizos from oxidative degradation.

## 1. Introduction

Agro-industries such as wineries and brewers have an economic relevance in the global market but also produce high quantities of wastes and by-products that could disrupt the environmental balance. There are many alternatives for reusing these materials, and their food utility has gained increasing interest. Their use as “natural” antioxidants could be one of the most efficient uses for these products. Their high content of phenolic compounds and their known antimicrobial power could lead to their use as substitutes for synthetic antioxidants.

For many years, the functional characteristics of many plant extracts have been evaluated because of their antioxidant and antimicrobial activity and their potential to replace synthetic antioxidants [1]. Grape (*Vitis vinifera*), green tea (*Camellia sinensis*) and chestnut (*Castanea sativa*) are of special interest due to their high content of phenolic compounds. Previous works reflect that grape seed extracts have antioxidant and antimicrobial activities in meat [2]; green tea was used to increase the shelf life of meat patties and pig liver pâté [3,4]; and the antioxidant activity of chestnut extract has also been investigated [3,5].

“Chorizo” is a typical dry fermented sausage from Spain. During the manufacturing process of chorizo, microbiological, chemical and physicochemical changes take place. In particular dehydration, fermentation of carbohydrates and acidification, development of color, lipolysis and fat autooxidation and proteolysis takes place [6]. Therefore, the use of antioxidants during processing aims to delay oxidation [3], allowing increase the shelf life of the product. Industries generally used synthetic antioxidants to control this process, such as butylated hydroxyanisole (BHA), butylated hydroxytoluene (BHT) and *tert*-butylhydroquinone (THBQ). However, the use of these synthetic compounds has been linked to health risks (carcinogenic potential) and current research tends for their replacement by natural antioxidants [5]. Thereby, increasing interest in natural antioxidants and a search for naturally occurring compounds with antioxidant activity has increased dramatically [7].

For the conservation and to extent the shelf life of the product, vacuum-packaging under refrigeration together with the use of natural antioxidants could be used to prevent major changes during storage, especially removing oxygen, which is the main cause of food oxidation [8]. To our knowledge, not many studies regarding the effect of natural antioxidants on the oxidation stability of dry ripened sausage “chorizo” were found in the related literature [5]. In addition, not much data about the dose to be used of natural antioxidants is available, only on rosemary and tea extracts in sausages and patties, respectively [9,10]. Therefore, the aim of this study was to evaluate the dose effect of the addition of natural antioxidants (tea, chestnut, grape seed and beer extracts) on physicochemical, microbiological changes and on oxidative stability of dry-cured “chorizo”, comparing their effect with a synthetic antioxidant (BHT), as well as knowing the effect of these natural extracts during the storage under vacuum conditions.

## 2. Experimental Section

### 2.1. Extraction of Natural Antioxidants

Grape seed extract (GRA) and chestnut extract (CHE) were prepared as previously was indicated in Lorenzo *et al.* [5], while the extraction of green tea extract (TEA) was carried out as described in Lorenzo *et al.* [3]. Beer residue was provided by Hijos de Rivera S.A. (A Coruña, Spain). This residue was used as source of polyphenolic compounds. This suspension residue comes from process of boiling of the must, where the temperature is maintained at 102 °C for 90 min. The objective of boiling is to obtain the necessary density, evaporating the spare water; the sterilizing the must and extracting and dissolving the wanted elements of hops. Lots of 4 L of this residue was transferred to XAD-16 amberlite column (Sigma-Aldrich, Spain). A glass column (7 cm Ø in × 40 cm height) filled with XAD-16 amberlite was equilibrating with distillate water to separate polyphenolic compounds. Four liters of distillate water was poured on the column to remove impurities; later, three liters of ethanol was used to elute polyphenols. This volume was evaporated until 200 mL (or until ethanol was completely removed) remained. Subsequently, the residue was lyophilized using a freeze-dryer (Kinetics EZ-Dryer, Stone Ridge, NY, USA). This lyophilized extract (raw extract of beer by-product) rendered 9.41 ± 34.0 g/L. This extract was subsequently used for the evaluation of the antioxidant capacity.

### 2.2. Determination of Antioxidant Capacity

#### 2.2.1. Determination of Total Phenolic Content

The total phenolic content was determined using the Folin-Ciocalteu Reagent (FCR) with gallic acid as a standard. Readings were performed at 765 nm and were compared with a standard curve of gallic acid, being the total phenolic content expressed as mg of gallic acid equivalent per g of freeze dried solid (mg GAE/g). Analyses were performed in triplicate.

#### 2.2.2. Trolox Equivalent Antioxidant Capacity (TEAC)

This assay is based on the scavenging of ABTS radical (2,2-azinobis-(3-ethyl-benzothiazoline-6-sulphonate)), observed as a decolorization of blue-green color at 734 nm. The radical scavenging capacity was compared with that of Trolox and results were expressed as g of Trolox equivalent per g of freeze dried solid.

#### 2.2.3. β-Carotene Bleaching Assay

The β-carotene (βC) bleaching assay described by Marco [11] was modified for use with microplates. Absorbance readings (470 nm) were taken at regular intervals in a ThermoFisher Scientific microplate reader until β-carotene was decolored (about 3 h). The antioxidant activity coefficient (AAC) and EC_50_ value (g/L) were calculated as previously described in Lorenzo *et al.* [3] for each antioxidant extract.

#### 2.2.4. α,α-Diphenyl-β-Picrylhydrazyl (DPPH) Radical Scavenging Activity

The antioxidant activity was determined with DPPH as a free radical, using microplate. Antioxidant solutions (10 μL) were added in triplicate to 200 μL of a 60 μM solution of DPPH in 70% ethanol. The decrease in absorbance was followed at 515 nm every 5 min until the reaction reached a plateau (about 2 h). The EC_50_ and BHT equivalent activity were calculated as explained above.

### 2.3. Manufacture of Dry-Cured Sausages

Four batches (20 units per batch, 3 per ripening time) of dry-cured sausage “chorizo”: Control (CON), BHT, grape seed (GRA), chestnut (CHE), green tea (TEA) and beer extracts (BER) were manufactured in the pilot plant of the Meat Technology Center of Galicia. Sausages were manufactured using the primal cuts of shoulder (85%) and pork back fat (15%) from Celta pig breed. The lean and the pork back fat were ground through a 6 mm diameter mincing plate in a refrigerated mincer machine (La Minerva, Bologna, Italy). Mixture was vacuum minced in a vacuum mincer machine (Fuerpla, Valencia, Spain) for 3 min with 5 g/kg of NaCl, 20 g/kg of sweet paprika, 3 g/kg of spicy paprika, 0.5 g/kg of garlic and 200 mg/kg of BHT for BHT batch, 0.05–0.2–1 g/kg of natural extracts. No starter culture was added. The meat mixture was maintained at 3–5 °C for 24 h and then was stuffed into pig gut (diameter 32–34 mm) to obtain an average final sausage weight of 150 g. After stuffing, the sausages were conditioned for two days at 7 °C and 85% of relative humidity. The sausages were transferred to a drying-ripening chamber where they were kept for 48 days at 12 °C and 75%–80% of relative humidity. Below the samples were packed under vacuum conditions five months at 4 °C. Analyses were carried out at 0, 4, 19 and 48 days of ripening time and at 2, 4 and 7 months of vacuum-packaged. The studied parameters were determined in duplicate for every sampling point.

### 2.4. Determination of pH, Moisture Content, Water Activity and Color Parameters

The pH of samples was measured using a pH-meter (HI 99163, Hanna Instruments, Eibar, Spain) equipped with a glass probe for penetration. Moisture percentage was determined by oven drying (Memmert UFP 600, Schwabach, Germany) at 105 °C until constant weight [12], and calculated as sample (5 g) weight loss. Water activity was determined using a Fast-lab (Gbx, Romans sur Isére, Cédex, France) water activity meter, previously calibrated with sodium chloride. A portable colorimeter (Konica Minolta CM-600d, Osaka, Japan) with pulsed xenon arc lamp, 0° viewing angle geometry and 8 mm aperture size, was used to estimate meat color in the CIELAB space: lightness, (L*); redness, (a*); yellowness, (b*). Each sausage piece was cut (2 cm) and the color of the slices was measured three times for each analytical point.

### 2.5. Determination of Lipid Oxidation

Lipid stability was evaluated through TBARS index according to the method proposed by Targladis *et al.* [13]. Briefly, the dry-cured sausage sample (10 g) was dispersed in distilled water (50 mL) and homogenized in an Ultra-Turrax (Ika T25 basic, Staufen, Germany) for 2 min. The homogenate was carried to a distillation system with HCl 4N (2.5 mL) and distilled water (47.5 mL) until recover 50 mL of distilled. The filtrate (5 mL) was reacted with a 0.02 M thiobarbituric acid (TBA) solution (5 mL) and incubated in a water bath at 96 °C for 40 min. The absorbance was measured at 538 nm. Thiobarbituric acid reactive substances (TBARS) values were calculated from a standard curve of malonaldehyde with 1,1,3,3-tetraethoxipropane (TEP) and expressed as mg MDA/kg sample.

### 2.6. Determination of Texture Profile Analysis

Texture profile analysis (TPA) was measured by compressing to 50% with a compression probe of 19.85 cm^2^ of surface contact in seven dry-cured sausage slices of 2 cm using a texture analyzer (TA.XTplus, Stable Micro Systems, Vienna Court, UK). Force-time curves were recorded at a crosshead speed of 1 mm/s. Hardness (kg), cohesiveness, springiness (mm), gumminess (kg) and chewiness (kg × mm) were obtained. These parameters were obtained using the available computer software (Texture Exponent 32 (version 1.0.0.68), Stable Micro Systems, Vienna Court, UK).

### 2.7. Analysis of Free Fatty Acid Content

Total intramuscular lipids were extracted from 5 g of each minced sausage sample, according to Folch *et al.* [14] procedure. Free fatty acids were separated from fifty milligrams of the extracted lipids using aminopropyl (NH_2_) mini-columns as described by García-Regueiro *et al.* [15]. This fraction was transesterified with a solution of boron trifluoride (14%) in methanol, according to Carreau and Dubacq [16] and the FAMEs were stored at −80 °C until chromatographic analysis. Separation and quantification of FAMEs was determined following Lorenzo and Franco [17].

### 2.8. Microbial Analysis

For microbiological analysis, a 10 g sample of dry-cured sausage was aseptically weighted in a sterile plastic bag. Subsequently samples were homogenized with 90 mL of a sterile solution of 0.1% (w/v) peptone water (Oxoid, Unipath, Basingstoke, UK), containing 0.85% NaCl and 1% Tween 80 as emulsifier, for 2 min at 20–25 °C in a Masticator blender (IUL Instruments, Barcelona, Spain), thus making a 1/10 dilution. Serial 10-fold dilutions were prepared by mixing 1 mL of the previous dilution with 9 mL of 0.1% (w/v) sterile peptone water. Total viable counts (TVC) were enumerated in Plate Count Agar (PCA; Oxoid, Unipath Ltd., Basingstoke, UK) and incubated at 30 °C for 48 h; lactic acid bacteria (BAL) were determined on the Man Rogosa Sharpe medium Agar (Oxoid, Unipath Ltd., Basingstoke, UK) (pH 5.6) after an incubation at 30 °C for 5 days. After incubation, plates with 30–300 colonies were counted. The microbiological data were transformed into logarithms of the number of colony forming units (CFU/g).

### 2.9. Statistical Analysis

For the statistical analysis of the results, an analysis of variance (ANOVA) of one way using SPSS package (SPSS 19.0, Chicago, IL, USA) was performed for all variables considered in the study. The least squares mean (LSM) were separated using Duncan’s *t*-test. All statistical test of LSM were performed for a significance level *p* < 0.05.

## 3. Results and Discussion

### 3.1. Antioxidant Activity of the Extracts

GRA and TEA extracts showed the highest polyphenol content, mainly flavonoids and flavan-3-ols, which antioxidant activity has been demonstrated [2,18]. The major compounds found in TEA extracts was catechin, epicatechin, cinnamic acids and sugar-linked flavonols [19], while GRA extracts contained benzoic acids, monomer flavan-3-ols and oligomeric procyanidins [18]. The higher activity found in GRA extracts could be associated to its resveratrol content [20]. Regarding polyphenols in CHE and BER extracts, their concentration were significantly lower than the aforementioned natural extracts (28.9 and 89.0 *vs.* 373.0 and 390.0 mg GAE/g extract for BER and CHE *vs.* GRA and TEA extracts, respectively).

TEAC, DPPH and β-carotene were used to assess *in vitro* antioxidant activity of the natural extracts. These methods were directly related to polyphenol contents [4]. Therefore, GRA and TEA extracts showed the highest activities in these methods. In the case of TEAC, the aforementioned extracts displayed values 10 and 15-fold higher Trolox equivalent antioxidant capacity than CHE extract (0.27 *vs.* 2.93 and 4.06 g Trolox/g extract for CHE, GRA and TEA, respectively) and 20 and 40-fold higher than BER extract (0.09 g Trolox/g extract).

The scavenging activity found on DPPH radical showed the higher antioxidant power of BHT standard, followed by GRA and TEA extracts (1.80 and 2.18 g equivalent BHT/g extract, respectively). The values provided by BER and CHE were almost 4 and 8-fold lower than the aforementioned extracts (0.25 and 0.48 g equivalent BHT/g extract, respectively). The EC_50_ values obtained showed the same behavior, the powerful antioxidant activity of TEA and GRA (0.12 and 0.16 g extract/L, respectively) *vs.* BER and CHE extracts (data not shown).

β-carotene bleaching assay of the natural extracts showed similar activity values for CHE and TEA (0.53 and 0.69 g equivalent BHT/g extract, respectively), although GRA were the most active (1.28 g equivalent BHT/g extract) and BER the least active (0.25 g equivalent BHT/g extract). The EC_50_ values obtained displayed rather similar activity values for all the extracts (less than 0.10 g extract/L).

### 3.2. Effect of Antioxidants on Physicochemical Parameters during the Manufacturing Process and Vacuum Packaging

**Figure 1:**
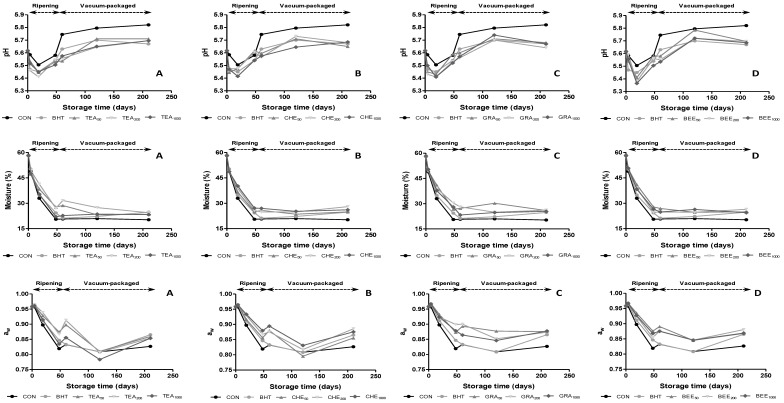
Evolution of pH values, moisture content and water activity in dry-cures sausages treated with butylated hydroxytoluene (BHT) and natural antioxidants during ripening and vacuum-packaged storage.

Changes occurred in pH, moisture content and water activity (a_w_) during the manufacturing process and vacuum-packing are given in Figure 1. Ripening time had a significant effect (*p* < 0.01) on pH values. During the first 19 days of ripening, pH values decreased from 5.62 to approximately 5.43 due to the production of lactic acid as a result of carbohydrate breakdown during fermentation [21] and the following increase can be produced by the liberation of peptides, amino acids and ammonia from proteolytic reactions [17]. Although at the end of ripening pH values of sausages were not affected (*p* > 0.05) by the addition of antioxidants, the highest pH values were observed in CHE_50_ and GRA_50_, followed by CON and BER_50_. These pH values were similar to those found in other varieties of sausages [22,23]. Regarding the evolution during vacuum packaging, there are not many studies that evaluate the influence of antioxidants on physicochemical parameters of dry-cured sausages. The trend is to continue growing slightly until 120 days to decreasing until the end of storage. The exception to this behavior is found in CON and TEA samples, which pH values continued increasing until the end of storage. Regarding dose effect, only TEA and GRA extracts presented significant (*p* < 0.05) differences on pH during ripening time and vacuum storage.

Moisture content was significantly (*p* < 0.01) affected by ripening time and addition of antioxidants, decreasing during the drying period as a result of moisture loss at high ripening temperature and low percentage of relative humidity (Figure 1). CON was the sample that presented the lowest value at the end of this stage with mean values of 20.6%, while samples manufactured with antioxidants presented higher values, in all cases above 22.0%. The moisture content and water activity followed similar behaviors because are variables that are intrinsically linked. As occurred with water content, the trend of water activity was to decrease over the time, obtaining significant differences during ripening time (*p* ≤ 0.001). As can be observed in Figure 1, two steps could be distinguished in its evolution. The first one represented a sharp decline in the values during ripening stage and the second one stabilization during the vacuum packaging until the end of the storage. No significant differences (*p* > 0.05) were observed in moisture and water activity among extracts depending on dose effect during ripening time and vacuum packaging.

Color parameters of the chorizo were significantly (*p* < 0.05) affected by ripening time and addition of antioxidants (Table 1). Regarding redness (a*), the trend was to decrease over time. Thereby, a significant (*p* < 0.01) color loss was observed during ripening time with values ranged between 27.6 and 21.2. This behavior could be due to the partial or total denaturation of nitrosomyoglobin caused by the production of lactic acid. Furthermore, a significant effect (*p* < 0.05) was also observed on redness with the use of antioxidants. Antioxidants were more effective in maintaining color, with higher values of redness in the samples manufactured with antioxidants. In this sense, samples manufactured with GRA_200_, CHE_50_, CHE_200_ and BHT presented higher a* values compared to CON batch; so that the addition of natural extracts improved the color stability, showing even better results than those showed by BHT.

During vacuum packaging, a slightly increase was observed in redness values. A similar behavior during packaging under vacuum was reported by Liaros *et al.* [24]. As happened during ripening, significant differences (*p* < 0.001) were also found with the addition of antioxidants. Regarding dose effect, significant differences (*p* < 0.01) were found for this color parameter in samples that contained TEA, CHE and GRA in their composition. During ripening, the highest values were obtained in the samples that containing a dose of 50 mg/kg in their composition, while vacuum packaging the dose more effective depends on the used extract. In this regards, Jayawardana *et al.* [25] showed the capacity of natural extracts to hold the color on pork sausages. As in the present study, the improving effects of extracts were concentration-dependent [1]. In this case, with the exception of samples that contain BER extract, a decrease of redness occurs at increasing levels of antioxidants.

**Table 1:** Evolution of color parameters of “chorizo” treated with BHT and natural antioxidants during ripening and vacuum-packaging.

	Days	CON	BHT	TEA			CHE			GRA			BER			*p*-Value	SEM
			200	50	200	1000	50	200	1000	50	200	1000	50	200	1000		
L*	4	43.30 ^e^	44.86 ^e^	43.79 ^d^	43.04 ^c^	42.52 ^c^	43.94 ^b^	42.51 ^b^	43.22 ^c^	44.61 ^d^	45.09 ^d^	42.55 ^c^	42.34 ^c^	42.33 ^c^	43.40 ^d^	0.186	0.23
	19	34.30 ^d,1^	40.05 ^d,3^	41.88 ^d,3,4^	43.19 ^c,4^	41.16 ^c,3,4^	42.49 ^b,4^	42.60 ^b,4^	41.62 ^c,3,4^	42.58 ^c,d,4^	41.85 ^d,3,4^	35.70 ^b,1,2^	36.49 ^b,2^	37.11 ^b,2^	37.21 ^c,2^	0.000	0.58
	48	30.46 ^c,1^	35.34 ^c,2–4^	38.98 ^c,4^	36.54 ^b,3,4^	32.03 ^a,b,1,2^	35.51 ^a,2–4^	34.88 ^a,2,3^	36.24 ^b,3,4^	36.96 ^a,b,3,4^	37.36^c,34^	34.70 ^b,2,3^	33.47 ^a,1–3^	32.13 ^a,1,2^	34.56 ^b,2,3^	0.008	0.49
	60	29.00 ^b,c,1,2^	31.68 ^a,3,4^	38.32 ^c,8^	39.23 ^b,c,8^	33.59 ^b,4,5^	34.48 ^a,5,6^	33.57 ^a,4,5^	34.60 ^a,b,5,6^	37.55 ^a,b,7,8^	35.94 ^b,c,6,7^	28.39 ^a,1^	31.81 ^a,3,4^	31.08 ^a,2,3^	29.24 ^a,1,2^	0.000	0.66
	120	28.63 ^b,1^	34.49 ^b,c,3–5^	35.58 ^b,5,6^	38.40 ^b,6,7^	32.93 ^b,2–5^	33.54 ^a,2–5^	35.06 ^a,4,5^	35.01 ^a,b,4,5^	39.68 ^b,c,7^	33.29 ^a,b,2–5^	30.81 ^a,1,2^	31.09 ^a,1–3^	31.64 ^a,1–4^	31.89 ^a,1–4^	0.000	0.59
	210	26.80 ^a,1^	32.84 ^a,b,2,3^	31.85 ^a,2,3^	31.41 ^a,2^	30.38 ^a,2^	31.78 ^a,2,3^	34.86 ^a,3^	32.34 ^a,2,3^	34.71 ^a,3^	30.52 ^a,2^	30.24 ^a,2^	31.08 ^a,2^	32.04 ^a,2,3^	30.04 ^a,2^	0.003	0.41
	*p-value*	0.000	0.000	0.000	0.005	0.000	0.001	0.005	0.001	0.001	0.001	0.000	0.000	0.000	0.000		
	*SEM*	1.82	1.39	1.20	1.27	1.41	1.43	1.20	1.22	1.05	1.52	1.43	1.24	1.24	1.48		
a*	4	26.17 ^c,d,2–5^	27.30 ^a,b,5^	26.57 ^a,3–5^	25.82 ^a,1–5^	24.90 ^b,c,1,2^	26.07^a,2345^	25.44 ^a,1–4^	24.43 ^a,b,1^	26.60 ^a,3–5^	26.94 ^a,4,5^	24.53 ^1^	26.24 ^2–5^	25.26 ^1–3^	26.17 ^2–5^	0.007	0.18
	19	23.58 ^a–c,1^	28.43 ^b,c,3,4^	29.43 ^b,c,4,5^	29.21 ^b,4,5^	26.90 ^c,2,3^	29.15 ^b,4,5^	29.09 ^b,c,4,5^	27.02 ^b,c,2,3^	30.51 ^b,c,5^	29.80 ^b,4,5^	24.37 ^1^	26.97 ^2,3^	26.80 ^2,3^	26.69 ^2^	0.000	0.39
	48	21.20 ^a,1^	25.44 ^a,b,2–6^	28.18 ^a,b,6^	24.82 ^a,2–6^	22.23 ^a,b,1,2^	25.93 ^a,3–6^	25.46 ^a,2–6^	24.27 ^a,b,1–5^	27.67 ^a,b,5,6^	27.28 ^a,4–6^	23.71 ^1–4^	24.87 ^2–6^	22.73 ^1–3^	24.44 ^1–5^	0.010	0.43
	60	22.61 ^a,b,1^	26.57 ^a,b,3^	31.88 ^c,5^	32.25 ^c,5^	26.06 ^c,2,3^	26.77 ^a,b,3^	26.79 ^a–c,3^	25.54 ^a–c,2,3^	29.59 ^a–c,4^	29.53 ^b,4^	23.02 ^1^	25.89 ^2,3^	26.97 ^3^	24.22 ^1,2^	0.000	0.56
	120	23.94 ^a–c,1^	30.42 ^c,4,5^	30.73 ^b,c,4 5^	32.33 ^c,5^	26.07 ^c,1–3^	29.16 ^b,3–5^	29.90 ^c,4,5^	28.07 ^c,2–4^	31.91 ^c,5^	26.06 ^a,1–3^	23.11 ^1^	24.60 ^1^	24.88 ^1,2^	25.23 ^1,2^	0.000	0.61
	210	24.93 ^b–d^	24.93 ^a^	26.17 ^a^	25.07 ^a^	20.85 ^a^	25.00 ^a^	25.85 ^a,b^	22.86 ^a^	27.83 ^a,b^	26.23 ^a^	22.80	23.59	25.12	25.25	0.066	0.39
	*p-value*	0.007	0.030	0.010	0.001	0.016	0.033	0.051	0.046	0.026	0.005	0.805	0.229	0.126	0.117		
	*SEM*	0.59	0.62	0.67	0.99	0.72	0.54	0.60	0.59	0.60	0.47	0.39	0.43	0.51	0.32		
b*	4	32.50 ^c^	35.10 ^e^	35.28 ^c^	33.95 ^b^	33.93 ^c^	34.11 ^c^	34.22 ^b^	32.84 ^c^	35.85 ^c^	37.01 ^d^	33.15 ^c^	34.04 ^d^	33.13 ^c^	33.92 ^d^	0.249	0.30
	19	21.59 ^b,1^	30.22 ^d,3^	30.91 ^b,c,3^	33.06 ^b,3^	31.13 ^c,3^	32.40 ^c,3^	33.77 ^b,3^	31.89 ^c,3^	33.43 ^c,3^	32.99 ^c,3^	23.36 ^b,1,2^	25.22 ^c,2^	26.13 ^b,2^	25.06 ^c,2^	0.000	0.80
	48	19.62 ^a,b,1^	23.71 ^c,1–4^	30.73 ^b,c,5^	23.01 ^a,1–4^	20.55 ^b,1,2^	25.15 ^b,1–5^	26.28 ^a,2–5^	25.73 ^b,2–5^	28.50 ^b,4,5^	27.52 ^b,3–5^	24.02 ^b,1–4^	22.31 ^b,c,1–3^	21.86 ^a,1–3^	23.30 ^b,c,1–4^	0.014	0.66
	60	17.09 ^a,1,2^	20.17 ^a,b,2–4^	27.84 ^b,7,8^	30.03 ^b,8^	21.57 ^b,3–5^	22.32 ^a,b,4–6^	21.64 ^a,3–5^	21.88 ^a,b,3–5^	25.28 ^a,b,6,7^	24.10 ^a,5,6^	16.69 ^a,1^	18.81 ^a,b,1–3^	19.97 ^a,2–4^	16.68 ^a,1^	0.000	0.77
	120	17.82 ^a,b,1^	23.45 ^b,c,2,3^	26.36 ^b,3,4^	28.91 ^b,4^	22.37 ^b,1–3^	23.58 ^b,2,3^	26.30 ^a,3,4^	25.45 ^b,3,4^	28.97 ^b,4^	21.45 ^a,1–3^	17.73 ^a,1^	19.28 ^a,b,1,2^	20.23 ^a,1,2^	20.19 ^a,b,1,2^	0.001	0.76
	210	17.81 ^a,b^	17.11 ^a^	19.75 ^a^	19.87 ^a^	15.43 ^a^	18.98 ^a^	20.62 ^a^	17.76 ^a^	21.48 ^a^	21.73 ^a^	17.21 ^a^	16.76 ^a^	19.38 ^a^	18.73 ^a^	0.072	0.42
	*p-value*	0.000	0.000	0.003	0.002	0.000	0.000	0.013	0.003	0.002	0.000	0.000	0.000	0.001	0.000		
	*SEM*	1.55	1.84	1.51	1.60	1.96	1.66	1.72	1.65	1.49	1.77	1.77	1.76	1.51	1.73		

^a–e^ Mean values in the same column (same antioxidant in different weeks) with different letter presented significant differences; ^1–8^ Mean values in the same row (different antioxidant in the same week) with different number presented significant differences; SEM: standard error of mean; Batches: CON: control; BHT: tert-butyl-4-hydroxytoluene; TEA: tea; CHE: chestnut; GRA: grape seed and BER: beer extracts.

The values of lightness (L*) decreased significantly (*p* < 0.01) until the end of the storage. This behavior could be due as a consequence of moisture losses [17]. Therefore, a significant correlation was found with moisture (*r* = 0.87, *p* < 0.01) and a_w_ (*r* = 0.80, *p* < 0.01). These results were lower than those found by other authors [6,24]. During ripening time the values ranged from 43.8 to 30.5, being CON the samples that showed the lowest values. As in previous studies conducted with these extracts [5], the samples that contained extracts in their composition showed higher L* values. At the end of the ripening process, the samples that contain TEA_50_, TEA_200_, GRA_50_ and GRA_200_ in their formulation were those showed the highest values, even improving the results obtaining for BHT (38.98, 36.54, 36.96, and 37.36 *vs.* 35.34, respectively). The same behavior was observed during vacuum packaging, the decline in the values continued, reaching values between 34.9 and 26.8, and the values were higher in samples with added extracts, being CHE_200_ the samples that showed the highest values. Regarding dose effect, significant differences (*p* < 0.05) were found for this color parameter in samples that contained GRA in their composition.

### 3.3. Effect of Antioxidants on TPA Analysis during the Manufacturing Process and Vacuum Packaging

The evolutions of TPA parameters (hardness, springiness, chewiness, gumminess and cohesiveness) during ripening and vacuum-packaged are shown in Table 2. The major changes take place during fermentation when the pH declines and the myofibrillar proteins aggregate leading to gel formation. Thereby, a significant increase (*p* < 0.05) in hardness, chewiness and gumminess was observed during the first 48 days of ripening, to remain stable until the end of storage. In the case of springiness, the values decreased during ripening to remain constant during vacuum-packaged. Drying is a major factor affecting binding and rheological properties [26]. In fact, a significant correlation (*p* < 0.01) were found between moisture and water activity and TPA parameters. In the case of hardness, chewiness, gumminess and cohesiveness these correlations with moisture (*r* = −0.67, *r* = −0.48, *r* = −0.75, *r* = −0.26, respectively) and water activity were negative (*r* = −0.59, *r* = −0.38, *r* = −0.65, *r* = −0.31, respectively), so an increase in these parameters during ripening time was due to a decrease in moisture and water activity. In addition, other research have shown that polyphenolic compounds are able to react with thiol groups in meat protein to form covalent thiol-quinone adducts [27]. Specifically, it has been hypothesized that polyphenolic compounds from green tea extract can alter the textural properties of Bologna type sausages [28], so especially at elevated concentrations phenolics compounds could interact with the protein thiols to modify water holding capacity and other textural parameters.

The addition of antioxidants significantly (*p* < 0.001) decreased hardness values. The lower results were found in the samples treated with TEA_200_, CHE_200_ and GRA_50_, followed by the samples treated with BHT and BER extracts. The values obtained for CON samples at the end of ripening were similar to those obtained in other studies [6] and lower to the results reported by González-Fernández *et al.* [26] in Galician chorizos. On the other hand, significant differences (*p* < 0.05) were found in TEA, CHE and GRA depending on the dose. The lower results of hardness were obtained with the dose of 200 mg/kg both during ripening and vacuum-packaging.

**Table 2:** Evolution of textural properties of “chorizo” treated with BHT and natural antioxidants during ripening and vacuum-packaging.

	Days	CON	BHT	TEA			CHE			GRA			BER			*p*-Value	SEM
			200	50	200	1000	50	200	1000	50	200	1000	50	200	1000		
***Hardness (kg)***	4	2.59 ^a,2–4^	2.10 ^a,1–3^	2.47 ^a,2,3^	1.49 ^a,1^	2.01 ^a,1–3^	1.78 ^a,1,2^	2.10 ^a,1–3^	1.96 ^a,1–3^	2.70 ^a,3–5^	2.27 ^a,1–3^	3.98 ^a,6^	3.36 ^a,4–6^	3.46 ^a,,56^	3.69 ^a,6^	0.000	0.15
	19	8.66 ^b,7^	7.77 ^b,6,7^	4.27 ^b,1–4^	5.61 ^b,3–6^	5.35 ^b,2–5^	3.97 ^b,1–3^	2.76 ^a,1^	2.49 ^a,1^	3.12 ^a,1,2^	3.51 ^a,1–3^	8.73 ^b,7^	6.29 ^a,4–6^	5.08 ^a,2–5^	7.20 ^b,5–7^	0.000	0.42
	48	17.50 ^d,7^	9.84 ^b,3–5^	6.89 ^c,1^	6.56 ^b,1^	8.60 ^c,2,3^	7.01 ^c,1^	6.97 ^b,1^	7.59 ^b,1,2^	7.47 ^b,1,2^	8.91 ^b,c,2–4^	10.30 ^c,4,5^	10.30 ^b,4,5^	12.00 ^b,c,6^	10.52 ^b,c,5^	0.000	0.55
	60	15.77 ^c,5^	9.98 ^b,3^	7.78 ^c,1,2^	6.82 ^b,1^	8.52 ^c,2^	7.37 ^c,1,2^	7.60 ^b,1,2^	7.68 ^b,1,2^	7.78 ^b,1,2^	7.89 ^b,1,2^	11.17 ^c,3^	10.91 ^b,3^	13.93 ^c,4^	10.24 ^b,3^	0.000	0.50
	120	16.07 ^c,d,7^	9.36 ^b,3–6^	10.70 ^d,5,6^	6.30 ^b,1^	8.74 ^c,2–5^	7.45 ^c,1–3^	8.21 ^b,1–4^	7.30 ^b,1–3^	7.12 ^b,1,2^	9.81 ^c,4–6^	11.23 ^c,6^	11.29 ^b,6^	10.22 ^b,4–6^	9.78 ^b,4–6^	0.000	0.47
	210	16.07 ^c,d,5^	9.01 ^b,12^	11.27 ^d,2,3^	8.58 ^c,1,2^	9.57 ^c,1,2^	9.22 ^d,1,2^	7.78 ^b,1^	8.24 ^b,1^	9.55 ^c,1,2^	9.66 ^c,1,2^	12.83 ^d,3,4^	14.44 ^c,4,5^	13.64 ^c,3–5^	13.84 ^c,3–5^	0.000	0.52
	*p-value*	0.000	0.001	0.000	0.000	0.005	0.000	0.000	0.000	0.000	0.000	0.000	0.001	0.000	0.005		
	*SEM*	1.73	0.85	0.96	0.67	0.84	0.75	0.77	0.79	0.76	0.91	0.86	1.12	1.24	0.99		
***Springiness (mm)***	4	0.42 ^b,4^	0.38 ^b,3,4^	0.34 ^1–3^	0.36 ^2,3^	0.29 ^1^	0.33 ^1,2^	0.36 ^d,2,3^	0.31 ^1,2^	0.31 ^1,2^	0.30 ^1,2^	0.30 ^1^	0.30 ^1,2^	0.31 ^1,2^	0.29 ^1^	0.001	0.01
	19	0.31 ^a^	0.35 ^b^	0.28	0.31	0.30	0.33	0.32 ^b,c^	0.33	0.32	0.31	0.35	0.32	0.32	0.36	0.390	0.01
	48	0.31 ^a^	0.28 ^a^	0.31	0.29	0.27	0.28	0.29 ^a,b^	0.30	0.29	0.34	0.30	0.35	0.33	0.32	0.246	0.01
	60	0.30 ^a,1–4^	0.27 ^a,1,2^	0.32 ^2–5^	0.31 ^2–5^	0.26 ^1^	0.28 ^1–3^	0.28 ^a,1–3^	0.34 ^4,5^	0.30 ^1–4^	0.31 ^2–5^	0.29^1234^	0.31 ^2–5^	0.36 ^5^	0.33 ^3–5^	0.016	0.01
	120	0.31 ^a^	0.29 ^a^	0.31	0.30	0.30	0.27	0.31 ^b,c^	0.30	0.30	0.30	0.30	0.29	0.29	0.28	0.666	0.01
	210	0.31 ^a,1–3^	0.29 ^a,1^	0.29 ^1^	0.29 ^1^	0.30 ^1,2^	0.32 ^1–4^	0.33 ^c,d,1–4^	0.31 ^1–3^	0.32 ^1–3^	0.31 ^1–3^	0.33 ^1–4^	0.35 ^2–4^	0.37 ^4^	0.36 ^3,4^	0.035	0.01
	*p-value*	0.000	0.001	0.074	0.318	0.190	0.376	0.007	0.410	0.625	0.649	0.218	0.424	0.130	0.069		
	*SEM*	0.01	0.01	0.01	0.01	0.01	0.01	0.01	0.01	0.01	0.01	0.01	0.01	0.01	0.01		
***Chewiness (kg × mm)***	4	0.25 ^a,3^	0.14 ^a,1,2^	0.14 ^a,1,2^	0.18 ^a,1^	0.10 ^a,1^	0.10 ^a,1^	0.15 ^a,1,2^	0.09 ^a,1^	0.12 ^a,1,2^	0.09 ^a,1^	0.20 ^a,2,3^	0.16 ^a,1,2^	0.19 ^a,2,3^	0.17 ^a,1–3^	0.009	0.01
	19	0.97 ^b,4^	0.62 ^b,3^	0.19 ^a,1^	0.17 ^a,b,1,2^	0.31 ^a,b,1,2^	0.22 ^a,1,2^	0.21 ^a,1,2^	0.18 ^a,1^	0.20 ^a,1,2^	0.24 ^a,1,2^	0.86 ^b,4^	0.43 ^b,2,3^	0.33 ^a,b,1,2^	0.63 ^b,3^	0.000	0.05
	48	1.29 ^c,6^	0.74 ^b,2,3^	0.65 ^b,1,2^	0.27 ^b,c,1^	0.64 ^b,c,1,2^	0.53 ^b,1^	0.55 ^b,1^	0.66 ^b,1,2^	0.63 ^b,1,2^	0.97 ^b,4,5^	0.88 ^b,3,4^	1.08 ^d,5^	1.04 ^c,d,4,5^	0.89 ^b,3–5^	0.000	0.05
	60	1.11 ^b,c,5^	0.65 ^b,1,2^	0.75 ^b,c,1–3^	0.30 ^c,,12^	0.56 ^a–c,1^	0.61 ^b,1,2^	0.58 ^b,c,1^	0.85 ^c,2–4^	0.69 ^b,1,2^	0.79 ^b,1–4^	0.85 ^b,2–4^	1.02 ^d,4,5^	1.41 ^d,e,6^	0.97 ^b,3–5^	0.000	0.05
	120	1.29 ^c,4^	0.75 ^b,1–3^	1.01 ^d,3^	0.32 ^c,1,2^	0.80 ^c,1–3^	0.51 ^b,1^	0.76 ^b,c,1–3^	0.62 ^b,1,2^	0.66 ^b,1,2^	0.83 ^b,2,3^	0.86 ^b,2,3^	0.83 ^c,2,3^	0.81 ^b,c,1–3^	0.77 ^b,1–3^	0.003	0.04
	210	1.16 ^b,c,1–3^	0.72 ^b,1^	0.84 ^c,1,2^	0.27 ^c,1^	0.84 ^c,1,2^	0.87 ^c,1,2^	0.82 ^c,1,2^	0.73 ^b,1^	0.98 ^c,1–3^	0.87 ^b,1,2^	1.33 ^c,2–4^	1.48 ^e,3,4^	1.69 ^e,4^	1.47 ^c,3,4^	0.005	0.07
	*p-value*	0.000	0.004	0.000	0.018	0.037	0.001	0.002	0.000	0.002	0.006	0.000	0.000	0.004	0.001		
	*SEM*	0.13	0.07	0.10	0.02	0.09	0.08	0.08	0.09	0.09	0.11	0.10	0.13	0.17	0.12		
***Gumminess (kg)***	4	0.58 ^a,3–5^	0.35 ^a,1–3^	0.39 ^a,1–4^	0.27 ^a,1^	0.35 ^a,1–3^	0.30 ^a,1,2^	0.43 ^a,1–5^	0.28 ^a,1^	0.41 ^a,1–4^	0.30 ^a,1,2^	0.67 ^a,5^	0.54 ^a,2–5^	0.61 ^a,4,5^	0.59 ^a,3–5^	0.013	0.03
	19	3.00 ^b,5^	1.80 ^b,3,4^	0.66 ^a,1,2^	0.77 ^a,1,2^	1.04 ^a,b,1,2^	0.69 ^a,1,2^	0.68 ^a,1,2^	0.54 ^a,1^	0.58 ^a,1^	0.78 ^a,1,2^	2.41 ^b,4,5^	1.33 ^a,2,3^	1.05 ^a,1,2^	1.85 ^b,3,4^	0.000	0.15
	48	4.26 ^c,6^	2.62 ^c,3–5^	2.07 ^b,1–3^	1.68 ^b,1^	2.34 ^c,2–4^	1.87 ^b,1,2^	1.89 ^b,1,2^	2.19 ^b,c,1–3^	2.16 ^b,1–3^	2.86 ^b,4,5^	2.93 ^c,5^	3.14 ^b,5^	3.15 ^b,c,5^	2.84 ^b,c,4,5^	0.000	0.13
	60	3.79 ^c,5^	2.44 ^c,1,2^	2.35 ^b,1^	2.05 ^b,1^	2.21 ^b,c,1^	2.12 ^b,1^	2.11 ^b,1^	2.52 ^d,1–3^	2.30 ^b,1^	2.48 ^b,1,2^	2.93 ^c,2–4^	3.26 ^b,4^	3.93 ^c,d,5^	3.01 ^c,3,4^	0.000	0.12
	120	4.23 ^c,6^	2.60 ^c,1–5^	3.30 ^d,5^	2.01 ^b,1,2^	2.69 ^c,2–5^	1.93 ^b,1^	2.46 ^b,1–4^	2.08 ^b,1,2^	2.20 ^b,1–3^	2.83 ^b,3–5^	2.94 ^c,4,5^	2.95 ^b,4,5^	2.82 ^b,3–5^	2.81 ^b,c,3–5^	0.000	0.12
	210	3.76 ^c,3–5^	2.47 ^c,1^	2.92 ^c,1–3^	2.25 ^b,1^	2.70 ^c,1,2^	2.66 ^c,1,2^	2.48 ^b,1^	2.36 ^c,d,1^	3.09 ^c,1–4^	2.74 ^b,1,2^	3.98 ^d,3–5^	4.28 ^c,5^	4.52 ^d,5^	4.14 ^d,4,5^	0.001	0.16
	*p-value*	0.000	0.000	0.000	0.002	0.012	0.000	0.001	0.000	0.000	0.000	0.000	0.000	0.000	0.002	
	*SEM*	0.44	0.25	0.33	0.23	0.29	0.25	0.25	0.27	0.30	0.32	0.30	0.39	0.44	0.34	
***Cohesiveness***	4	0.23 ^a,b^	0.17 ^a^	0.16 ^a^	0.10 ^a^	0.18 ^a^	0.17 ^a^	0.21 ^a^	0.16 ^a^	0.15 ^a^	0.14 ^a^	0.17 ^a^	0.16 ^a^	0.17 ^a^	0.16 ^a^	0.256	0.01
	19	0.34 ^c,5^	0.23 ^b,2–4^	0.18 ^a,1,2^	0.23 ^a,1^	0.20 ^a,1,2^	0.18 ^a,1,2^	0.22 ^a,1–^	0.22 ^b,1–4^	0.19 ^a,1,2^	0.20 ^a,1,2^	0.27 ^b,4^	0.22 ^b,1–4^	0.21 ^a,1–3^	0.26 ^b,3,4^	0.000	0.01
	48	0.24 ^b^	0.27 ^b^	0.30 ^c^	0.48 ^b^	0.28 ^b^	0.27 ^b^	0.28 ^a,b^	0.30 ^c^	0.29 ^b^	0.32 ^b^	0.29 ^b^	0.30 ^c^	0.26 ^b^	0.27 ^b^	0.260	0.01
	60	0.24 ^b,1^	0.25 ^b,1^	0.30 ^c,3–5^	0.64 ^b,3–5^	0.26 ^b,1,2^	0.29 ^b,2–4^	0.28 ^a,b,1–3^	0.33 ^d,5^	0.30 ^b,2–5^	0.32 ^b,4,5^	0.26 ^b,1,2^	0.30 ^c,3–5^	0.29 ^b,2–4^	0.30 ^b,2–5^	0.001	0.01
	120	0.27 ^b^	0.28 ^b^	0.31 ^c^	0.61 ^b^	0.31 ^b^	0.26 ^b^	0.30 ^b^	0.29 ^c^	0.31 ^b^	0.29 ^b^	0.26 ^b^	0.27 ^c^	0.28 ^b^	0.29 ^b^	0.102	0.01
	210	0.23 ^a,b,1^	0.28 ^b,1–4^	0.26 ^b,1,2^	0.66 ^b,1–3^	0.28 ^b,1–4^	0.29 ^b,2–5^	0.32 ^b,4,5^	0.29 ^c,2–5^	0.33 ^b,5^	0.29 ^b,2–5^	0.31 ^b,3–5^	0.30 ^c,2–5^	0.33 ^c,5^	0.30 ^b,2–5^	0.006	0.01
	*p-value*	0.002	0.027	0.000	0.003	0.003	0.002	0.030	0.000	0.000	0.006	0.010	0.001	0.001	0.002		
	*SEM*	0.01	0.01	0.02	0.07	0.02	0.02	0.01	0.02	0.02	0.02	0.02	0.02	0.02	0.02		

^a−e^ Mean values in the same column (same antioxidant in different weeks) with different letter presented significant differences; ^1−7^ Mean values in the same row (different antioxidant in the same week) with different number presented significant differences.

Gumminess and chewiness were significantly (*p* < 0.05) increased during ripening time, reaching mean values of 4.26 kg and 1.29 kg × mm, respectively. This increase indicated that gumminess changed from short to pasty gummy through ripening. The highest values were observed in CON samples, while the lowest were found in the samples treated with TEA and GRA extracts. Within the dose effect, significant differences (*p* < 0.05) were found among samples treated with different concentrations of TEA, CHE and GRA extracts. In the case of TEA, the samples treated with 200 mg/kg were that showed the lowest values, while a dose of 50 mg/kg were enough in CHE and GRA samples. Chewiness values indicated that sausages became tougher during ripening period. As occurred with gumminess, the lowest values were found in the samples treated with TEA extract. Significant differences (*p* < 0.05) were found among batches during ripening time. However, regarding dose effect, only significant differences were found in samples treated with TEA extract (0.65 *vs.* 0.27 *vs.* 0.64 kg × mm in TEA_50_, TEA_200_ and TEA_1000_, respectively).

Springiness values have been related to the elastic properties of sausages [29]. The values decreased during ripening to remain constant during vacuum-packaged, but only in CON and samples treated with BHT and CHE_200_ this decreased was significant (*p* < 0.01). This result could be also due to water removal during the ripening period. No significant differences (*p* > 0.05) were observed among treatments and the end of ripening, but these differences were significant (*p* < 0.05) at the end of the vacuum-packaging (Table 2).

### 3.4. Effect of Antioxidants on Oxidative Stability during the Manufacturing Process and Vacuum Packaging

**Figure 2:**
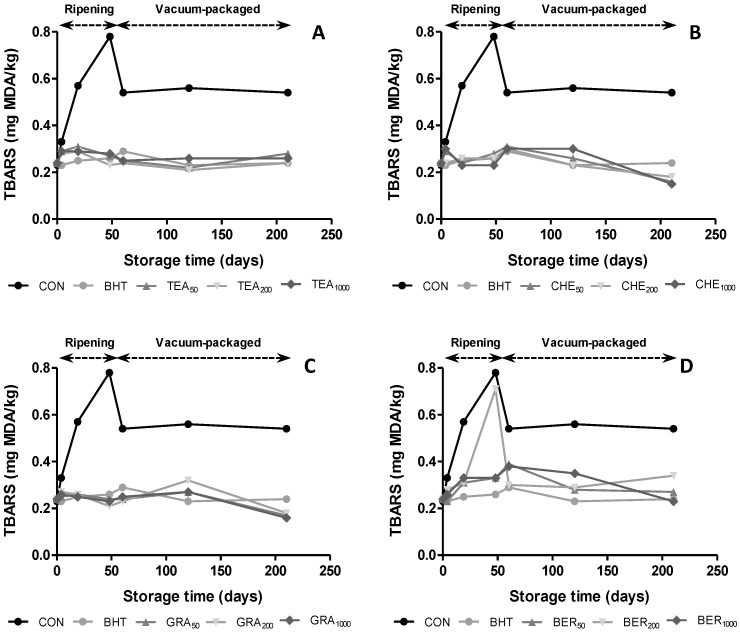
Evolution of thiobarbituric acid reactive substances (TBARS) in dry-cures sausages treated with BHT and natural antioxidants during ripening and vacuum-packaged storage.

The influence of antioxidants on oxidative stability during the manufacturing process and vacuum packaging was evaluated as TBARS index (Figure 2). Significant changes (*p* < 0.05) were detected in TBARS values among samples during storage time. According to other authors [5,10], CON batches showed more intense lipid oxidation. Thereby, samples with contained antioxidants in their composition showed values below 0.4 mg MDA/kg, showing that these extracts reduced lipid oxidation of the dry-cured sausage. The maximum TBARS values were observed at the end of ripening time (values between 0.23 to 0.78 mg MDA/kg at 0 and 48 days, respectively) followed by a decline until day 60, to remain constant to the end of vacuum packaging. The values found during ripening and vacuum packaging were higher than those found by other authors [6,30] and similar to those found in other dry-cured sausages [17]. The levels obtained during storage period were significantly (*p* < 0.05) lower than the limit (2.0 mg MDA/kg) which is accepted as deterioration level [31].

Regarding the effect of the addition of antioxidants, the results obtained were equal or even better to those found with BHT. Thereby, the samples treated with CHE and GRA reached mean values of 0.17 mg MDA/kg at the end of vacuum packaging, while the samples that contained BHT showed values of 0.24 mg MDA/kg. These results are in agreement with previously published studies [4], which reported higher effectiveness of natural products compared to synthetic antioxidants and suggesting the possibility of using these extracts as replacers of commercial compounds. Within dose effect, no significant differences (*p* > 0.05) were found among samples on the same extract, so that the lower concentration of natural extract would be sufficient to improve the results obtained in CON samples.

### 3.5. Effect of Antioxidants on Microbial Counts during the Manufacturing Process and Vacuum Packaging

Changes in the microbial populations, TVC, LAB and mold/yeasts, during the manufacturing process and vacuum packaging of dry-cured sausage “chorizo” are shown in Table 3. The initial TVC, LAB and mold/yeasts counts ranged from 10^3^ to 10^5^ CFU/g (data not shown). Significant differences (*p* < 0.05) in microbial counts were detected among batches and during ripening and storage period. TVC counts increased from 5.17 to 8.62 log_10_ CFU/g (*p* < 0.001) during the first 19 days of ripening, remaining stable until the end of ripening (reaching values of 8.72 log_10_ CFU/g). TVC counts continued to increase up to day 60, to proceed decreasing gradually during vacuum storage. Among batches, significant differences (*p* < 0.001) were found with the addition of antioxidants. At the end of ripening process, samples that contained extracts in their composition showed lower TVC counts than CON. Excluding CHE_200_, natural extracts showed lower results, getting to improve the results of BHT (8.12 log_10_ CFU/g *vs.* counts below 8 log_10_ CFU/g in TEA_200_, CHE_200_, GRA_1000_, BER_200_ and BER_1000_). Regarding dose effect, significant differences (*p* < 0.001) were found for all batches studied. Increase the level of natural antioxidant usually decreased the TVC counts. In fact, samples manufactured with GRA_1000_ showed lower values than GRA_50_, GRA_200_ and CON (7.91 log_10_ CFU/g *vs.* 8.14, 8.15 and 8.55 log_10_ CFU/g, respectively). In the other extracts, the dose of 200 mg/kg showed the lowest values (7.97, 7.88 and 7.95 log_10_ CFU/g for TEA, CHE and BER, respectively).

**Table 3:** Evolution of TVC, BAL and mold/yeast of “chorizo” treated with BHT and natural antioxidants during ripening and vacuum-packaging.

	Days	CON	BHT	TEA			CHE			GRA			BER			*p*-Value	SEM
			200	50	200	1000	50	200	1000	50	200	1000	50	200	1000		
***TVC***	4	6.35 ^a,8^	6.06 ^a,4,5^	6.34 ^a,8^	5.88 ^a,2^	6.12 ^a,5,6^	6.11 ^a,5,6^	5.97 ^a,3^	5.94 ^a,3^	6.14 ^a,6,7^	6.09 ^a,4–6^	6.18 ^a,7^	6.12 ^a,5,6^	5.72 ^a,1^	6.04 ^a,4^	0.000	0.03
	19	8.62 ^e,8^	8.38 ^e,4^	8.42 ^d,4,5^	8.59 ^c,7,8^	8.51 ^d,6^	8.55 ^f,6,7^	8.46 ^d,5^	8.22 ^d,2^	8.19 ^c,1,2^	8.32 ^e,3^	8.31 ^e,3^	8.21 ^d,2^	8.16 ^c,1^	8.58 ^e,7,8^	0.000	0.03
	48	8.55 ^d,8^	8.12 ^c,5–7^	8.06 ^b,4^	7.97 ^b,3^	8.08 ^c,4,5^	8.10 ^d,4–6^	7.88 ^c,1^	8.72 ^f,9^	8.14 ^c,6,7^	8.15 ^d,7^	7.91 ^c,1,2^	8.10 ^c,4–6^	7.95 ^b,2,3^	7.99 ^c,3^	0.000	0.04
	60	9.07 ^f,8^	8.24 ^d,2^	8.30 ^c,2^	8.70^d,6^	8.55 ^d,4^	8.28 ^e,2^	8.84 ^e,7^	8.62 ^e,5^	8.43 ^d,3^	8.65 ^f,5,6^	8.27 ^e,2^	8.48 ^e,3^	8.46 ^d,3^	8.15 ^d,1^	0.000	0.05
	120	7.61 ^c,1^	7.92 ^b,3^	8.00 ^b,4^	7.90 ^b,3^	7.99 ^c,4^	7.99 ^c,4^	7.87 ^c,3^	7.80 ^b,2^	7.91 ^b,3^	7.61 ^b,1^	8.12 ^d,6^	8.06 ^c,5^	8.13 ^c,6^	7.92 ^b,3^	0.000	0.03
	210	7.47 ^b,1^	8.15 ^c,5^	9.92 ^e,6^	7.91 ^b,3,4^	7.88 ^b,3,4^	7.92 ^b,3,4^	7.73 ^b,2^	8.15 ^c,5^	7.93 ^b,4^	7.81 ^c,2,3^	7.85 ^b,3,4^	7.86 ^b,3,4^	7.93 ^b,4^	7.93 ^b,4^	0.000	0.11
	*p-value*	0.000	0.000	0.000	0.000	0.000	0.000	0.000	0.000	0.000	0.000	0.000	0.000	0.000	0.000		
	*SEM*	0.37	0.24	0.32	0.28	0.25	0.24	0.27	0.28	0.23	0.25	0.22	0.23	0.28	0.24		
***LAB***	4	6.62 ^a,2^	6.91 ^a,6^	6.87 ^a,6^	6.62 ^a,2,3^	7.07 ^a,7^	7.11 ^a,7^	6.54 ^a,1^	6.71 ^a,4^	6.78 ^a,5^	6.89 ^a,6^	6.88 ^a,6^	6.55 ^a,1^	6.67 ^a,3,4^	6.78 ^a,5^	0.000	0.03
	19	7.97 ^c,6,7^	7.90 ^b,c,3,4^	7.95 ^c,5,6^	7.93 ^b,c,4,5^	8.00 ^b,7–9^	8.00 ^b,7–9^	7.95 ^d,5,6^	7.84 ^b,2^	8.01 ^b,c,8,9^	7.90 ^c,3,4^	7.86 ^a,b,2,3^	7.97 ^b,6–8^	7.76 ^b,1^	8.03 ^c,9^	0.000	0.04
	48	8.12 ^d,6,7^	8.07 ^d,5,6^	8.01 ^c,4,5^	8.02 ^c,d,45^	8.18 ^c,7^	7.97 ^b,3,4^	7.80 ^c,d,2^	8.57 ^c,8^	7.93 ^b,3^	7.96 ^c,3,4^	7.70 ^a,b,1^	8.16 ^b,7^	8.03 ^b,4,5^	7.84 ^b,2^	0.000	0.04
	60	11.08 ^f,8^	7.96 ^c,d,1,2^	7.91 ^c,1^	8.06 ^d,3^	8.00 ^b,2,3^	8.20 ^b,4,5^	8.51 ^e,6^	8.72 ^c,7^	8.23 ^c,5^	8.49 ^d,6^	8.14 ^b,4^	8.05 ^b,3^	8.44 ^c,6^	8.18 ^d,4,5^	0.000	0.15
	120	8.32 ^e,2–4^	8.32 ^e,2–4^	7.95 ^c,1–4^	7.85 ^b,1–3^	8.51 ^d,3,4^	8.50 ^b,3,4^	7.34 ^b,1^	8.00 ^b,1–4^	9.71 ^d,6^	7.58 ^b,c,1,2^	8.66 ^b,4,5^	8.03 ^b,1–4^	8.00 ^b,1–4^	9.28 ^e,5,6^	0.000	0.13
	210	7.45 ^b^	7.75 ^b^	7.54 ^b^	7.90 ^b^	8.22 ^c^	7.87 ^b^	7.73 ^c^	7.94 ^b^	8.09 ^b,c^	7.32 ^a,b^	7.68 ^a,b^	7.69 ^b^	8.11 ^b,c^	7.95 ^b,c^	0.104	0.06
	*p-value*	0.000	0.000	0.001	0.000	0.000	0.038	0.000	0.000	0.000	0.002	0.042	0.003	0.000	0.000		
	*SEM*	0.50	0.13	0.13	0.15	0.14	0.14	0.18	0.20	0.26	0.16	0.18	0.17	0.17	0.22		
***Mold/Yeast***	4	6.09 ^d,6^	5.78 ^a,3^	5.86 ^d,3,4^	5.67 ^b,2^	5.80 ^b,3^	6.00 ^c,5^	6.44 ^c,7^	5.56 ^c,1^	5.89 ^d,4^	5.89 ^e,4^	6.48 ^e,7^	5.78 ^b,3^	5.94 ^c,4,5^	6.13 ^d,6^	0.000	0.05
	19	7.74 ^e,4^	7.10 ^b,1,2^	7.69 ^e,3,4^	7.52 ^c,2–4^	7.16 ^c,1,2^	7.10 ^d,1,2^	7.60 ^d,3,4^	7.28 ^d,1–3^	7.31 ^e,1–4^	7.07 ^f,1^	7.38 ^f,1–4^	7.05 ^c,1^	7.75 ^e,4^	7.67 ^e,3,4^	0.006	0.06
	48	6.17 ^d,6^	5.61 ^a,1^	5.91 ^d,4^	5.71 ^b,2^	5.96 ^b,4^	5.81 ^c,3^	5.91 ^b,c,4^	5.73 ^c,2^	5.79 ^d,3^	5.61 ^c,1^	6.19 ^d,6^	6.12 ^b,5^	6.15 ^d,5,6^	6.24 ^d,7^	0.000	0.04
	60	5.92 ^c,9^	5.58 ^a,6^	5.20 ^c,3^	5.78 ^b,7,8^	5.51 ^b,5^	5.28 ^a,b,4^	5.23 ^b,3^	5.31 ^b,4^	5.08 ^b,2^	4.93 ^a,1^	5.78 ^c,7,8^	5.89 ^b,9^	5.74 ^b,7^	5.82 ^c,8^	0.000	0.06
	120	5.58 ^b,5^	5.21 ^a,3–5^	4.53 ^b,1,2^	5.05 ^a,b,2–5^	5.08 ^b,2–5^	5.50 ^b,4,5^	4.37 ^a,1^	4.60 ^a,1–3^	4.89 ^a,1–4^	5.15 ^b,2–5^	5.43 ^a,4,5^	5.23 ^a,3–5^	5.58 ^b,5^	5.53 ^b,4,5^	0.004	0.08
	210	4.49 ^a,2^	5.12 ^a,2,3^	2.79 ^a,1^	4.66 ^a,2,3^	3.25 ^a,1^	5.04 ^a,2,3^	4.48 ^a,2^	4.74 ^a,2,3^	5.32 ^c,2,3^	5.48 ^c,2,3^	5.57 ^b,3^	5.23 ^a,2,3^	4.67 ^a,2,3^	5.23 ^a,2,3^	0.000	0.16
	*p-value*	0.000	0.043	0.000	0.003	0.000	0.000	0.000	0.000	0.000	0.000	0.000	0.001	0.000	0.000		
	*SEM*	0.36	0.22	0.45	0.28	0.36	0.20	0.35	0.27	0.24	0.21	0.20	0.19	0.28	0.24		

^a−f^ Mean values in the same column (same antioxidant in different weeks) with different letter presented significant differences; ^1−9^ Mean values in the same row (different antioxidant in the same week) with different number presented significant differences; SEM: standard error of mean; Batches: CON: control; BHT: tert-butyl-4-hydroxytoluene; TEA: tea; CHE: chestnut; GRA: grape seed and BER: beer extracts.

The lactic acid bacteria (LAB) counts showed significant (*p <* 0.05) differences during ripening and storage. A rapid increase in the LAB population was observed during the first 19 days of fermentation, increasing counts from 4.7 to 8.0 log_10_ CFU/g. Until the end of fermentation and ripening process the counts remained constant, reaching values of between 8.57 and 7.70 log_10_ CFU/g. The samples than contained CHE_1000_ in their formulation showed the highest values, followed by CON, TEA_1000_ and BER_50_, that presented similar values around 8.15 log_10_ CFU/g. Except for CON samples, the trend during vacuum packaging was to decreased slightly (33% *vs.* mean values of 5.1%).

Dose effect showed significant (*p <* 0.05) differences among batches during ripening process on LAB counts. GRA and BER extracts showed a decrease in the population of LAB with the increase of the concentration, obtaining lower values in GRA_1000_ (7.70 *vs.* 7.93 and 7.96 for GRA_1000_, GRA_50_ and GRA_200_, respectively) and BER_1000_ (7.84 *vs.* 8.16 and 8.03 for BER_1000_, BER_50_ and BER_200_, respectively). In contrast, samples treated with TEA and CHE showed lower counts for the minor dose (Table 3). As happened with the aforementioned microbial groups, mold and yeasts counts increased rapidly during the first 19 days of ripening, from 3.4 to 7.7 log_10_ CFU/g, to decrease slightly until the end of ripening process with values ranged between 6.2 and 5.61 log_10_ CFU/g.

### 3.6. Effect of Antioxidants on Free Fatty Acid Content during the Manufacturing Process and Vacuum Packaging

The free fatty acid (FFA) content of the different batches expressed as mg of fatty acid/g of fat is shown in Table 4 and Table 5. The predominated fatty acids both the end of ripening time and the end of storage at vacuum-packaging were monounsaturated fatty acids (MUFA), followed by saturated fatty acids (SFA) and polyunsaturated fatty acids (PUFA). These results are in agreement with other studies conducted in dry-ripened “chorizo” [6], being oleic, linoleic, palmitic, stearic and palmitoleic acids the predominated ones in the stages of ripening.

The free fatty acid content during ripening and vacuum packaging showed a gradual and significant release of these compounds as a result of lipolysis. Significant differences (*p <* 0.05) were detected among samples at the end of ripening time. In all cases, CON samples showed lower release values than those obtained for the samples treated with antioxidants. MUFAs were the FFA that showed the highest increases, greater than 70%. Oleic acid was the predominant fatty acid presented in all the batches, with values that ranged between 49% and 55%. These values reached the highest levels between days 4 and 19 of ripening, being the samples treated with BHT, TEA_1000_, CHE_200_ and BER_1000_ that reached the maximum release values. Unlike other authors [32], the percentages of oleic acid continued to increase until the end of ripening and during vacuum storage.

**Table 4:** Evolution of free fatty acid composition (mg/100g) of “chorizo” treated with BHT and natural antioxidants during ripening and vacuum-packaging.

	Days	CON	BHT	TEA			CHE			GRA			BER			*p*-Value	SEM
			200	50	200	1000	50	200	1000	50	200	1000	50	200	1000		
***C16:0***	4	274.05 ^a,b^	119.86 ^a^	280.66 ^a^	233.36 ^a^	219.68 ^a^	207.05 ^a^	247.90 ^a^	159.44 ^a^	250.42 ^a^	215.51 ^a^	223.47 ^a^	244.31	194.55	167.68	0.711	13.10
	19	166.96 ^a^	393.41 ^b^	350.09 ^a^	289.73 ^a^	281.74 ^a^	324.98 ^a,b^	414.83 ^a^	227.25 ^a^	227.42 ^a^	210.42 ^a^	303.13 ^a^	271.75	288.36	297.27	0.227	16.63
	48	169.33 ^a,1^	353.28 ^b,3,4^	375.84 ^a,4^	322.48 ^a,2–4^	507.68 ^b,5^	331.06 ^a,b,2–4^	341.90 ^a,2–4^	396.63 ^b,4^	317.90 ^a,2–4^	373.45 ^a,4^	270.06 ^a,2,3^	330.28 ^2–4^	255.06 ^2^	314.01 ^2–4^	0.000	15.12
	210	426.16 ^b^	700.61 ^c^	743.92 ^b^	1227.20 ^b^	689.07 ^c^	801.26 ^b^	936.76 ^b^	820.64 ^c^	749.68 ^b^	1006.20 ^b^	691.26 ^b^	600.02	594.27	673.13	0.082	44.54
	*p-value*	0.052	0.002	0.056	0.004	0.002	0.083	0.056	0.000	0.006	0.009	0.012	0.090	0.060	0.079		
	*SEM*	43.88	79.57	75.17	158.85	71.85	97.41	111.39	97.54	82.71	128.05	73.48	60.72	64.69	80.04		
***C16:1***	4	29.32 ^a^	1.64 ^a^	27.99 ^a^	23.72 ^a^	14.56 ^a^	26.28 ^a^	26.27 ^a^	11.72 ^a^	26.66 ^a^	13.88 ^a^	16.66 ^a^	18.07 ^a^	17.05 ^a^	8.47 ^a^	0.438	2.17
	19	14.16 ^a^	53.91 ^b^	39.91 ^a^	34.66 ^a^	32.77 ^a^	35.07 ^a^	50.31 ^a^	23.83 ^a^	26.46 ^a^	21.55 ^a^	35.22 ^a^	29.97 ^a^	34.88 ^a^	37.55 ^a^	0.081	2.39
	48	19.26 ^a,1^	49.30 ^b,2,3^	54.19 ^a,3^	43.30 ^a,2,3^	89.21 ^b,4^	48.80 ^a,2,3^	43.91 ^a,2,3^	55.45 ^b,3^	46.49 ^a,2,3^	57.41 ^b,3^	38.35 ^a,2^	47.80 ^a,2,3^	36.01 ^a,2^	43.39 ^a,2,3^	0.000	2.95
	210	76.10 ^b,1^	103.16 ^c,1,2^	106.19 ^b,1,2^	167.12 ^b,3^	112.31 ^b,1,2^	102.71 ^b,1,2^	123.62 ^b,1–3^	109.10 ^c,1,2^	116.46 ^b,1,2^	149.79 ^c,2,3^	97.67 ^b,1^	91.46 ^b,1^	97.90 ^b,1^	103.37 ^b,1,2^	0.038	5.09
	*p-value*	0.009	0.003	0.010	0.001	0.001	0.006	0.030	0.000	0.013	0.000	0.009	0.019	0.032	0.026		
	*SEM*	9.60	13.85	11.72	22.18	15.27	11.56	15.07	14.33	14.60	20.50	11.92	11.11	12.45	13.90		
***C18:0***	4	143.67	89.44 ^a^	143.01	114.11 ^a^	104.97 ^a^	107.01	130.30	91.68 ^a^	121.10 ^a^	129.85 ^a^	124.49 ^a,b^	135.63	111.36	102.55	0.900	5.87
	19	92.34	179.57 ^b^	166.29	139.56 ^a^	142.81 ^a,b^	166.92	203.95	130.09 ^a^	124.16 ^a^	122.16 ^a^	154.38 ^a,b^	142.90	157.77	167.49	0.269	6.87
	48	69.20 ^1^	116.22 ^a,1,2^	123.21 ^2^	96.85 ^a,1,2^	176.92 ^b,3^	101.52 ^1,2^	106.23 ^1,2^	133.60 ^a,2,3^	115.99 ^a,1,2^	144.12 ^a,2,3^	104.71 ^a,1,2^	137.68 ^2,3^	99.33 ^1,2^	126.71 ^2^	0.021	5.54
	210	149.17	223.33 ^b^	259.89	461.58 ^b^	240.33 ^c^	345.60	351.84	280.00 ^b^	232.18 ^b^	395.92 ^b^	245.44 ^b^	215.10	211.30	321.35	0.552	22.98
	*p-value*	0.262	0.006	0.254	0.055	0.014	0.296	0.226	0.003	0.015	0.067	0.109	0.269	0.131	0.256		
	*SEM*	16.60	20.44	25.51	62.53	19.71	49.61	45.73	27.68	19.22	48.29	23.56	16.33	19.62	41.52		
***C18:1n9c***	4	529.15 ^a^	213.02 ^a^	468.86 ^a^	443.31 ^a^	338.88 ^a^	438.33	459.15	339.35 ^a^	511.66 ^a^	409.07 ^a^	452.25 ^a^	491.65 ^a^	400.20 ^a^	344.88 ^a^	0.465	22.37
	19	432.85 ^a,1^	973.25 ^b,3,4^	853.91 ^a,2–4^	733.01 ^a,1–4^	688.11 ^b,1–3^	799.28 ^2–4^	1043.61 ^4^	624.51 ^a,b,1,2^	645.37 ^a,b,1,2^	551.32 ^a,b,1,2^	671.73 ^a,1–3^	682.09 ^a,1–3^	696.65 ^b,1–3^	784.79 ^b,2–4^	0.029	34.50
	48	405.56 ^a,1^	1030.46 ^b,4^	855.82 ^a,2–4^	875.07 ^a,2–4^	1278.56 ^c,5^	858.61 ^2–4^	784.46 ^2,3^	991.82 ^b,3,4^	916.95 ^b,2–4^	1072.72 ^b,4^	803.00 ^a,b,2,3^	952.68 ^b,2–4^	732.70 ^b,2^	894.76 ^b,2–4^	0.000	38.41
	210	899.38 ^b^	1350.71 ^c^	1523.51 ^b^	2341.58 ^b^	1577.73 ^c^	1580.00	1791.42	1480.70 ^c^	1381.90 ^c^	2130.29 ^c^	1368.17 ^b^	1222.02 ^c^	1306.35 ^c^	1378.62 ^c^	0.126	85.05
	*p-value*	0.050	0.002	0.033	0.007	0.002	0.087	0.144	0.007	0.009	0.004	0.066	0.004	0.004	0.007		
	*SEM*	81.83	160.15	154.33	286.77	186.32	177.77	220.87	166.56	130.48	261.70	142.51	106.83	126.83	143.54		
***C18:2n6c***	4	305.74 ^b,5^	150.08 ^a,1^	270.91 ^3–5^	230.20 ^a,1–5^	186.19 ^a,1–3^	256.88 ^3–5^	273.12 ^4,5^	162.99 ^a,1,2^	226.89 ^a,1–5^	187.06 ^a,1–3^	246.44 ^2–5^	243.37 ^a,2–5^	194.95 ^a,1–4^	185.99 ^a,1–3^	0.015	9.79
	19	257.29 ^a,b,1^	397.66 ^b,3,4^	363.86 ^2–4^	351.91 ^b,1–4^	286.12 ^a,b,1,2^	340.63 ^1–4^	421.13 ^4^	301.49 ^b,1–3^	326.34 ^b,1–4^	261.83 ^a,b,1^	366.47 ^2–4^	373.61 ^b,2–4^	330.43 ^b,1–4^	372.16 ^b,2–4^	0.038	10.72
	48	174.46 ^a,1^	311.35 ^b,3,4^	250.16 ^2,3^	236.18 ^a,1–3^	427.53 ^b,c,5^	252.14 ^2,3^	205.00 ^1,2^	344.28 ^b,c,4^	302.38 ^a,b,3,4^	339.05 ^b,4^	311.37 ^3,4^	352.47 ^b,4^	308.50 ^b,3,4^	335.79 ^b,4^	0.000	13.06
	210	338.14 ^b^	335.41 ^b^	401.34	544.78 ^c^	438.75 ^c^	355.56	424.95	418.13 ^c^	367.91 ^b^	543.81 ^c^	364.37	404.98 ^b^	426.23 ^c^	464.93 ^b^	0.299	16.58
	*p-value*	0.056	0.005	0.176	0.003	0.022	0.351	0.256	0.004	0.048	0.001	0.240	0.042	0.010	0.019		
	*SEM*	25.70	35.50	28.96	49.16	41.94	24.62	46.38	35.96	21.22	50.99	23.61	24.99	32.28	40.08		
***C18:3n3***	4	15.81 ^b,3^	0.00 ^a,1^	10.18 ^a,b,2^	10.15 ^2^	0.00 ^a,1^	0.00 ^1^	9.51^2^	0.00 ^a,1^	3.80 ^a,1^	2.73 ^a,1^	0.00 ^1^	0.00 ^a,1^	0.00 ^a,1^	1.72 ^a,1^	0.000	1.03
	19	6.51 ^a^	17.06 ^b^	19.17 ^b^	13.94	11.99 ^a,b^	21.82	22.62	11.58 ^b^	11.91 ^b^	9.77 ^a,b^	23.90	14.21 ^b,c^	11.21 ^a,b^	16.73 ^b,c^	0.125	1.21
	48	0.00 ^a,1^	10.98 ^a,b,2^	5.52 ^a,1,2^	4.22 ^1,2^	20.54 ^b,3^	7.43 ^1,2^	6.13 ^1,2^	12.84 ^b,2,3^	8.32 ^a,b,1,2^	11.40 ^b,2^	8.07 ^1,2^	10.55 ^b,2^	6.10 ^a,1,2^	10.01 ^a,b,2^	0.024	1.03
	210	19.08 ^b^	22.13 ^b^	21.78 ^c^	30.41	22.83 ^b^	20.52	20.43	33.65 ^c^	22.22 ^c^	28.63 ^c^	22.37	21.06 ^c^	22.97 ^b^	22.60 ^c^	0.806	1.29
	*p-value*	0.009	0.055	0.184	0.001	0.054	0.161	0.217	0.001	0.009	0.002	0.092	0.006	0.026	0.022		
	*SEM*	2.96	3.44	3.05	3.72	3.73	4.16	3.32	4.64	2.66	3.66	4.30	2.96	3.40	3.11		
***C20:4n6***	4	9.54 ^a,b,1–3^	0.00 ^a,1^	17.79 ^3^	0.00 ^1^	8.36 ^a,1–3^	0.95 ^1^	16.32 ^2,3^	0.00 ^a,1^	0.00 ^a,1^	2.31 ^1^	0.00 ^a,1^	4.01 ^1,2^	0.00 ^a,1^	0.00 ^a,1^	0.035	1.38
	19	12.65 ^a,b^	20.26 ^b^	15.85	14.24	9.99 ^a,b^	15.12	19.53	8.93 ^a^	14.12 ^b^	8.81	23.98 ^c^	14.91	24.90 ^c^	18.77 ^b,c^	0.247	1.25
	48	0.00 ^a^	9.75 ^ab^	16.90	7.01	29.31 ^c^	9.28	9.52	6.23 ^a^	9.85 ^b^	8.98	12.27 ^b^	13.65	10.56 ^b^	11.27 ^a,b^	0.081	1.48
	210	21.43 ^b^	10.64 ^a,b^	13.57	15.63	19.21 ^b^	7.98	7.71	23.62 ^b^	14.31 ^b^	17.65	12.72 ^b^	19.12	23.29 ^c^	28.56 ^c^	0.062	1.38
	*p-value*	0.167	0.084	0.989	0.068	0.012	0.126	0.254	0.026	0.003	0.232	0.019	0.082	0.000	0.035		
	*SEM*	3.50	3.07	3.63	2.64	3.30	2.23	2.35	3.49	2.24	2.61	3.39	2.36	3.85	4.26		

^a–c^ Mean values in the same column (same antioxidant in different weeks) with different letter presented significant differences; ^1–5^ Mean values in the same row (different antioxidant in the same week) with different number presented significant differences; SEM: standard error of mean; Batches: CON: control; BHT: tert-butyl-4-hydroxytoluene; TEA: tea; CHE: chestnut; GRA: grape seed and BER: beer extracts.

**Table 5:** Evolution of main nutritional index (mg/100g) of “chorizo” treated with BHT and natural antioxidants during ripening and vacuum-packaging.

	Days	CON	BHT	TEA			CHE			GRA			BER			*p*-value	SEM
			200	50	200	1000	50	200	1000	50	200	1000	50	200	1000		
***SFA***	4	417.72	209.30 ^a^	423.68	347.47 ^a^	324.64 ^a^	314.06	378.20	251.12 ^a^	371.51 ^a^	345.36 ^a^	347.95 ^a^	379.94	305.91	270.24	0.780	18.54
	19	259.29	572.99 ^b^	516.38	429.29 ^a^	424.54 ^a^	491.91	618.78	357.33 ^b^	351.57 ^a^	332.58 ^a^	457.51 ^a^	414.64	446.13	464.77	0.241	23.34
	48	238.52^1^	469.49 ^b,2–4^	499.04 ^3,4^	419.32 ^a,2–4^	684.60 ^b,5^	432.58 ^2–4^	448.11 ^2–4^	530.23 ^c,4^	433.89 ^a,2–4^	517.57 ^a,4^	374.78 ^a,2,3^	467.96 ^2–4^	354.39 ^1,2^	440.72 ^2–4^	0.001	20.17
	210	605.07	957.29 ^c^	1039.82	1754.88 ^b^	962.22 ^c^	1195.06	1339.90	1152.74 ^d^	988.29 ^b^	1452.09 ^b^	974.31 ^b^	853.55	835.24	1032.75	0.189	68.56
	*p-value*	0.079	0.003	0.072	0.008	0.003	0.123	0.076	0.000	0.007	0.015	0.023	0.130	0.083	0.131		
	*SEM*	62.73	103.68	103.74	229.84	95.32	152.43	162.25	132.27	102.49	183.00	102.29	84.44	89.03	127.86		
***MUFA***	4	560.24 ^a^	214.66 ^a^	496.85 ^a^	467.03 ^a^	353.43 ^a^	464.60	485.42	351.06 ^a^	538.32 ^a^	422.95 ^a^	468.91 ^a^	509.72 ^a^	417.25 ^a^	353.35 ^a^	0.432	24.10
	19	447.01 ^a,1^	1027.17 ^b,3,4^	893.82 ^a,2–4^	767.66 ^a,1–4^	720.87 ^b,1–3^	834.35 ^2–4^	1093.92 ^4^	648.35 ^a,b,1,2^	671.83 ^a,b,1,2^	572.87 ^a,b,1,2^	706.95 ^a,1–3^	712.06 ^a,1–3^	731.53 ^a,b,1–3^	822.34 ^b,2–4^	0.031	36.77
	48	424.81 ^a,1^	1079.76 ^b,4,^	910.00 ^a,2–4^	918.37 ^a,2–4^	1368.99 ^c,5^	907.41 ^2–4^	828.37 ^2,3^	1047.27 ^b,3,4^	963.44 ^b,2–4^	1130.13 ^b,4^	841.36 ^a,b,2,3^	1000.48 ^b,2–4^	768.70 ^b,2^	938.15 ^b,2–4^	0.000	41.11
	210	990.17 ^b^	1469.62 ^c^	1650.85 ^b^	2546.22 ^b^	1712.08 ^c^	1709.73	1941.17	1608.25 ^c^	1522.29 ^c^	2313.74 ^c^	1481.89 ^b^	1327.59 ^c^	1419.73 ^c^	1498.93 ^c^	0.112	91.21
	*p-value*	0.039	0.001	0.028	0.006	0.001	0.077	0.133	0.005	0.008	0.003	0.057	0.004	0.005	0.008		
	*SEM*	93.32	174.94	168.48	314.60	204.09	193.11	239.88	183.01	148.03	287.04	156.57	119.33	141.34	159.35		
***PUFA***	4	331.08 ^b,5^	150.08 ^a.1^	298.87 ^4,5^	240.35 ^a,1–5^	194.55 ^a,1–3^	257.83 ^3–5^	298.93 ^4,5^	162.99 ^a,1,2^	230.68 ^a,1–4^	192.10 ^a,1–3^	246.44 ^2–5^	247.38 ^a,2–5^	194.95 ^a,1–3^	187.72 ^a,1–3^	0.008	11.29
	19	276.43 ^a,b,1^	434.97 ^c,4,5^	398.87 ^1–5^	380.08 ^b,1–5^	308.09 ^a,1–3^	377.57 ^1–5^	463.27 ^5^	321.99 ^b,1–4^	352.37 ^b,1–5^	280.42 ^b,1,2^	414.35 ^3–5^	402.73 ^b,2–5^	366.53 ^b,1–5^	407.65 ^b,c,3–5^	0.050	12.55
	48	174.46 ^a,1^	332.07 ^b,3,4^	272.57 ^2,3^	247.41 ^a,1–3^	477.38 ^b,5^	268.84 ^2,3^	220.64 ^1,2^	363.35 ^b,4^	320.54 ^a,b,3,4^	359.42 ^b,4^	331.71 ^3,4^	376.66 ^b,4^	325.15 ^b,3,4^	357.07 ^b,4^	0.000	14.81
	210	386.20 ^b^	372.15 ^b,c^	444.05	601.60 ^c^	490.66 ^b^	394.38	460.58	480.62 ^c^	414.98 ^b^	601.65 ^c^	403.06	451.71 ^b^	479.32 ^c^	522.59 ^c^	0.348	18.41
	*p-value*	0.050	0.005	0.226	0.002	0.019	0.284	0.256	0.002	0.030	0.001	0.169	0.030	0.008	0.018		
	*SEM*	32.42	41.13	33.59	56.28	49.13	30.73	51.10	43.67	26.91	58.12	30.73	30.60	39.74	48.02		
***P/S***	4	0.88	0.72 ^b^	0.74	0.72 ^b,c^	0.62	0.86	0.83 ^b^	0.65 ^b^	0.68	0.56	0.72 ^a,b^	0.66 ^a^	0.66	0.72	0.961	0.03
	19	1.07	0.77 ^b^	0.83	0.90 ^c^	0.73	0.78	0.76 ^b^	0.91 ^c^	1.02	0.91	0.91 ^b^	0.98 ^b^	0.83	0.91	0.597	0.03
	48	0.78 ^2–5^	0.72 ^b,1–5^	0.55 ^1,2^	0.60 ^a,b,1–3^	0.70 ^1–5^	0.62 ^1–3^	0.50 ^a,b,1^	0.69 ^b,1–4^	0.74 ^2–5^	0.71 ^1–5^	0.89 ^b,4,5^	0.81 ^a,b,3–5^	0.92 ^5^	0.82 ^3–5^	0.015	0.03
	210	0.64 ^5^	0.39 ^a,1–3^	0.43 ^1–4^	0.35 ^a,1,2^	0.51 ^1–5^	0.35 ^1,2^	0.34 ^a,1^	0.42 ^a,1–4^	0.42 ^1–4^	0.43 ^1–4^	0.42 ^a,1–4^	0.56 ^a,3–5^	0.60 ^4,5^	0.54 ^2–5^	0.036	0.02
	*p-value*	0.489	0.004	0.140	0.016	0.158	0.203	0.052	0.005	0.084	0.164	0.044	0.056	0.163	0.346		
	*SEM*	0.09	0.06	0.07	0.08	0.04	0.09	0.08	0.07	0.09	0.08	0.08	0.07	0.06	0.07		
***n6/n3***	4	20.25 ^b,2^	0.00 ^a,1^	30.45 ^2,3^	22.72 ^b,2^	0.00 ^a,1^	0.00 ^1^	37.77 ^2,3^	0.00 ^a,1^	59.71 ^b,4^	46.83 ^b,3,4^	0.00 ^a,1^	0.00 ^a,1^	0.00 ^a,1^	0.00 ^a,1^	0.000	4.00
	19	41.49 ^c,4^	24.72 ^b,1–3^	21.80 ^1–3^	26.23 ^c,1–3^	25.29 ^b,1–3^	19.39 ^1,2^	19.59 ^1,2^	26.85 ^c,1–3^	28.58 ^a,2,3^	29.09 ^a,2,3^	16.96 ^b,1^	27.36 ^c,1–3^	31.85 ^b,3,4^	23.66 ^b,1–3^	0.011	1.29
	48	0.00 ^a^	29.71 ^b^	48.95	41.03 ^d^	24.48 ^b^	38.01	45.20	29.04 ^c^	43.73 ^a,b^	31.36 ^a^	40.16 ^c^	34.75 ^d^	53.04 ^c^	35.76 ^b^	0.072	2.92
	210	18.85 ^b^	17.29 ^b^	19.50	18.46 ^a^	20.21 ^b^	19.06	22.21	13.16 ^b^	17.23 ^a^	19.61 ^a^	19.28 ^b^	20.50 ^b^	20.56 ^b^	22.05 ^b^	0.725	0.67
	*p-value*	0.000	0.014	0.105	0.000	0.018	0.066	0.578	0.007	0.048	0.027	0.005	0.000	0.001	0.009		
	*SEM*	5.56	4.46	5.05	3.22	4.10	5.66	6.73	4.56	6.61	3.94	5.53	4.92	7.34	5.06		

^a−c^ Mean values in the same column (same antioxidant in different weeks) with different letter presented significant differences; ^1−5^ Mean values in the same row (different antioxidant in the same week) with different number presented significant differences; SEM: standard error of mean; Batches: CON: control; BHT: tert-butyl-4-hydroxytoluene; TEA: tea; CHE: chestnut; GRA: grape seed and BER: beer extracts.

The increases were lower in PUFA and SFA, with values between 3%–145% and 8%–111%, respectively. Regarding PUFA, linoleic and arachidonic were the fatty acids that showed higher percentages of release. The samples treated with CHE_200_ showed a decrease (26%) during ripening that could be associated with the oxidation of PUFA and the decrease in the proportion of the long chain PUFAs such as arachidonic acid (42%) [33]. Within SFA, palmitic and stearic acid were the most abundant, with levels between 17.4%–22.8% and 6.2%–8.1%, respectively. Unlike what happened with oleic acid, stearic decreased toward the end of ripening in CON and in the samples treated with low dose of natural extracts (mean decreases of 52% *vs.* 12%), for increased again until the end of vacuum packaged.

**Figure 3:**
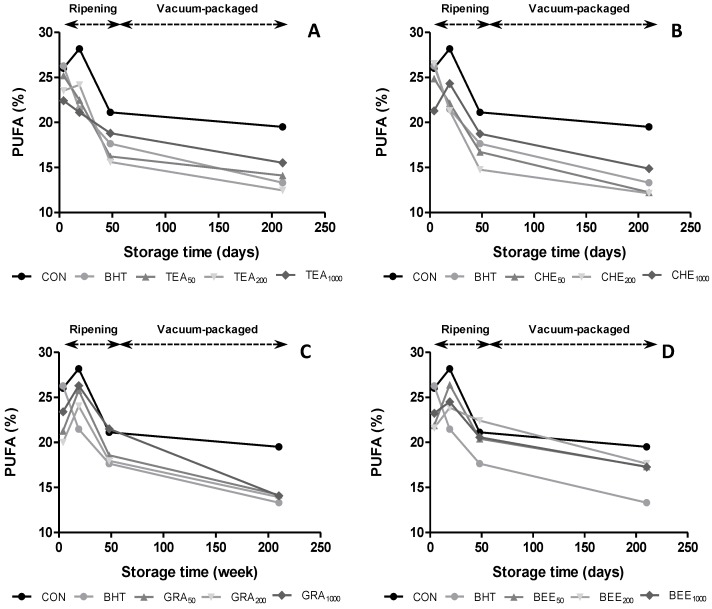
Evolution of PUFA content in dry-cures sausages treated with BHT and natural antioxidants during ripening and vacuum-packaged storage.

The amount of PUFA can be used as a measurement of the oxidative deterioration of meats, due to containing double bonds in the hydrocarbon chain being preferred substrates in oxidative reactions. Regarding oxidative stability, a significant correlation was found between PUFA and TBARS (*r* = −0.22, *p <* 0.05). As can be seen in Figure 3, the oxidative degradation of PUFA mainly occurred after day 19. At the end of ripening, we observed that the addition of GRA_1000_ and BER_200_ extracts protect chorizos from oxidative degradation since, higher amount of PUFA were observed in these treated samples than in CON (21.5% and 22.4% *vs.* 21.1%, respectively).

To assess the nutritional properties of IMF, the ratios PUFA/SFA and *n*-6/*n*-3 were determined (Table 5). The PUFA/SFA ratio showed mean values of 0.72, being CON and the samples treated with BER extract which showed the highest values. Significant differences (*p <* 0.05) were found among samples at the end of ripening and within storage time in samples treated with BHT and with higher doses of natural antioxidants (TEA_200_, CHE_200_, CHE_1000_ and GRA_1000_). In general, at the end of ripening the obtained values were within the typical values (0.5–0.7) of the Mediterranean diet [34] and lower than the FAO recommendations [35] for human diet (0.85), while at 210 days the values were lower (mean values lower than 0.46).

## 4. Conclusions

The addition of natural antioxidants changed the physicochemical properties of dry-cured sausages. The presence of antioxidants and the use of low concentrations improved color maintenance during ripening and under vacuum conditions. The results obtained for TBARS values showed that natural antioxidants matched or even improved the results obtained for BHT, with higher effectiveness for grape and chestnut extracts. The values of hardness decreased significant with the addition of antioxidants, obtaining the lowest values with the intermediate dose. Microbial counts were affected by the addition of antioxidants since lower counts were observed in sausages prepared with natural extracts. Free fatty acid content during ripening and vacuum packaging showed a gradual and significant release. The addition of grape and beer extracts protected sausages from oxidative degradation. Further analysis of how to affect the addition of natural extracts on sensory properties and volatile compounds of chorizo will be addressed.
